# Interrelationship between Interpersonal Interaction Intensity and Health Self-Efficacy in People with Diabetes or Prediabetes on Online Diabetes Social Platforms: An In-Depth Survey in China

**DOI:** 10.3390/ijerph17155375

**Published:** 2020-07-26

**Authors:** Zhihong Chen, Chaochuang Zhang, Guanhua Fan

**Affiliations:** Shantou University Medical College, Shantou 515041, China; 15zhchen5@stu.edu.cn (Z.C.); 15cczhang2@stu.edu.cn (C.Z.)

**Keywords:** online health community, diabetes mellitus, self-efficacy, peer assistance, group participation, health communication

## Abstract

*Objective:* The peer interaction–based online model has been influential in the recent development of diabetes management. This model “extends and innovates” the traditional mode of doctor–patient guidance, transforming it into a mode in which both doctor–patient guidance and patient–patient interaction coexist; this new mode has the added advantage of offering “extended continual intervention.” This study contributes to research on extending diabetes management models by investigating how patients with diabetes or prediabetes interact in online health communities, focusing on the interrelationship between self-efficacy characteristics and online participation during patient–patient interactions. *Methods:* In this cross-sectional study, participants with diabetes of various severities completed an electronic questionnaire, which was formulated with a revised classical scale. The questionnaire was disseminated through diabetes online health communities. Its content covered the respondent’s general condition, self-evaluation of their self-efficacy, and participation in online health communities, specifically with respect to factors such as the time spent in online information each day, the number of groups joined, and the extent of interaction in diabetes online health communities, etc. The main observation indicators were the participants’ self-efficacy, their extent of online participation, and the characteristics of online health communities. Descriptive statistics, chi-square test, linear trend estimation, and ordinal logistic regression were used to explore the relationship between the three indicators. *Results:* The self-efficacy scores ( $\bar{x}$ ± s) were 51.9 ± 9.12, and 59.1% of interviewed participants had self-efficacy scores greater than the mean. Overall, most participants (96%) considered online diabetes social platforms to be helpful. Groups differed with respect to interaction mode, which indicated that people with high self-efficacy tend to employ various modes of interaction. Participants with high self-efficacy were also more likely to live in cities (*p* < 0.05) and be married (*p* < 0.05) and tended to spend more time paying attention to group information (*p* < 0.05), spend more time viewing group information (*p* < 0.05), and have a greater degree of interaction with group members (*p* < 0.05). Information sources for the different grades of participants was primarily obtained from social media. *Conclusion:* Among people with diabetes, the frequency and intensity of online interaction might positively affect self-efficacy and, by implication, diabetes self-management. Diabetics with high self-efficacy also tend to have positive online interaction and adopt different ways of interaction. In addition, the diabetes information sources of the respondents mainly come from social networks, most of the respondents think that online social networking sites have a positive impact on diabetes self-management, which shows that social network plays an important role in diabetes information source of diabetics. However, the design of online health communities has room for improvement, specifically with respect to the provision of information that patients require. As an interesting side note, among people with diabetes or prediabetes, those who lived in urban area and were married, those who paid more attention to group information, and those who actively participated in interactions tended to have relatively high self-efficacy. The results suggest that people with diabetes have higher-quality self-care when they engage in online health community interactions; such benefits cannot be obtained from treatment in a hospital. In general, enhanced self-efficacy in people with diabetes enables them to more readily acquire diabetes-related knowledge. Online interaction with diabetics, who has the same experience, can not only get more information, but also have a sense of identity and belonging, which enhances self-efficacy and further urges them to actively participate in online interaction. Therefore, online health communities are an important supplement to the clinical treatment of diabetes mellitus and clinicians can take advantage of the educational function of online diabetes groups in their provision of tailored diabetes interventions and take into account the factors that affect the self-efficacy of diabetics (including the frequency and intensity of online interaction, age, marital status, residential area, etc.), to provide tailored diabetes interventions for diabetics. Such a use of online diabetes groups can strengthen diabetes self-management.

## 1. Introduction

Diabetes mellitus is a chronic disease that occurs worldwide. China currently has the largest population of people with diabetes in the world. Over the past three decades, the incidence of diabetes in China has risen sharply [1]. The latest China’s chronic disease and its risk factor monitoring (2013) published by Journal of American Medical Association (JAMA) in 2017 shows that among Chinese adults, the standardized prevalence of diagnosed and undiagnosed diabetes is 10.9% (95% confidence interval (CI), 10.4% −11.5%); pre-diabetes account for 35.7% (95% CI, 34.1% −37.4%). It is estimated that by 2020, the number of patients diagnosed with diabetes will be 155 million (10.9%), and pre-diabetes will be approximately 507 million (35.7%) [2].

Furthermore, with changes in the living and working practices of Chinese people—in particular, a sedentary lifestyle and affluent diet—the incidence of diabetes mellitus has been increasing, with urban areas being most affected. Although urban areas have a higher incidence of diabetes, rural areas have higher diabetes mortality [3]. Among adults in China, the prevalence of diabetes was approximately 10.9% in 2013, and that of prediabetes was approximately 35.7% [2]. Therefore, diabetes mellitus and its related complications constitute a heavy financial burden on Chinese families [4]. This indicates clear short-term effects of early control of blood glucose and its ability to reduce the risk of complications in doctor–nurse interventions. However, large-sample studies have demonstrated unsatisfactory glycemic control in patients with type 2 diabetes in China, with only 26.21–39.7% of participants achieving ideal blood glucose control and reaching the target of Hemoglobin A1c (HbA1c) < 7.0%; these findings were attributable to the discontinuity of medical intervention in the respective treatment mode and insufficient health literacy among patients [5,6]. Therefore, patient–patient interaction in diabetes control must be explored from multiple perspectives. In doing so, interventions can be adjusted to prevent diabetes-related complications.

Diabetes mellitus is a chronic non-communicable disease that must be controlled through long-term treatment and care; such care involves medication as well as management of the patient’s diet, exercise habits, and emotions. Therefore, self-management is crucial for people with diabetes; it is the cornerstone of diabetes treatment, especially in the control of blood glucose levels and reduction of diabetes-related complications [7,8]. Although patients with diabetes can acquire requisite knowledge and skills through health education, individuals differ considerably with respect to knowledge of and compliance with blood glucose control. Therefore, the present study explored whether self-efficacy affects the relationship between patient–patient interaction and blood glucose control by investigating whether online interaction is an effective strategy to improve diabetes self-management and self-efficacy in people with diabetes [9,10]. Self-efficacy, which was proposed by the US psychologist Bandura (2005), is a core concept in the theory of social learning and refers to an individual’s judgment regarding whether he or she is capable of undertaking a given behavior [11,12]. Studies have demonstrated that improved self-efficacy promotes behavioral change and improves self-management ability, quality of life, and confidence in coping with a disease. Similarly, measures for improving self-efficacy can also be applied to the intervention and treatment of chronic diseases such as diabetes [13,14,15,16]. Diabetes self-management refers to the voluntary behavior of diabetic patients in self-monitoring and regulating their blood glucose, preventing diabetic complications, and maintaining their overall mental and physical health. Research has suggested that the main factors influencing diabetes self-management are a patient’s diabetes knowledge, health education, self-efficacy, social support, communication skills, age, gender, and mental health status.

With the rapid development of Internet technology in China, many people with diabetes use online health communities as a platform for collecting and exchanging information. They use these platforms to share their experiences of self-management of diabetes. By using these platforms, individuals can obtain resources, information, and emotional support, resulting in better diabetes self-management through peer-to-peer education. Through online peer assistance, people with diabetes have improved their knowledge of diabetes self-management [17,18,19,20]. Confronted with common problems, people with diabetes engaging in an online interactive environment provide each other with material and emotional support, which reduces their self-efficacy in physical environments while greatly enhancing their self-efficacy in the online environment [21,22].

Coping strategies refer to the measures and countermeasures for coping with changes in a disease. Research has indicated that the self-care coping strategies adopted by patients with diabetes can be divided into active manager, passive follower, and nonconformist strategies. Specifically, an active manager actively adopts a healthy lifestyle and rather than perceiving diabetes as a disease, regards it as a condition that must be addressed. A passive follower prefers the fixed management mode to the flexible management mode, with fixed medication and mealtimes. Finally, nonconformists fail to adopt a healthy lifestyle or follow self-care programs, especially the diet and exercise courses that these programs prescribe [23,24]. Upon joining online health communities, patients with diabetes inevitably form social relationships and engage in online interaction. People in online health communities are vulnerable to the influence of others when exchanging information with them. Concomitantly, online health communities can affect the self-efficacy of people with diabetes, which partially affects their understanding of self-management. Therefore, online interaction partially affects individuals’ strategies for coping with diabetes [25].

As a new phenomenon, online health communities have become an “expandable and sustainable” health intervention for many patients with diabetes that supplements hospital treatment; such communities improve the psychological state and quality of life of patients [26]. At present, online health communities are widely used as the primary means of diabetes management in China, especially among young and middle-aged patients. The rapid development of this phenomenon has also accelerated the spread of other innovative practices, such as the sharing of inspirational stories, effective therapies, personal experience in blood glucose control and disease prevention, and medical word-of-mouth information. The theory of innovation diffusion proposed by Rogers (1995) follows that innovative activities can be diffused through communication channels and social networks [27]. The innovative activities generated by online health communities provide patients with diabetes with the resources that they require and increases the likelihood that these resources will be adopted and tested. Research findings have demonstrated that less complex and strongly operable network platforms can be used as a medium of interpersonal communication and mass communication. Such a medium is particularly crucial to innovation diffusion. Furthermore, user feedback can mitigate the negative effects resulting from the dissemination of harmful innovations through the platform. Therefore, online health communities for people with diabetes are most likely to facilitate the timely and effective transmission of new knowledge, provide an important platform for the innovative dissemination of both health knowledge and effective blood glucose control methods, and enable more accurate understanding of the disease itself and its management options [28,29,30].

Diabetes mellitus and its complications threaten the physical and mental health of patients; they also constitute an economic burden to the patient’s family and reduce the patient’s quality of life. Developments in health behavior theory have helped patients with diabetes to adopt better methods of coping with the progression of diabetes. Self-efficacy refers to the personal belief in one’s ability to achieve behavioral goals or cope with certain difficulties. Self-management emphasizes self-control by means of concept recognition, goal setting, self-monitoring, self-reinforcement, and practice. Many studies have demonstrated that self-efficacy is crucial for self-management. Recent research has inquired whether online diabetes groups, which are gaining popularity, can effectively function as a type of self-management intervention. To answer this question, we conducted an in-depth survey on members of online health groups with diabetes or prediabetes; we investigated the interrelationship between their intensity of interpersonal interaction and health self-efficacy. Our results can aid people with diabetes or prediabetes in their diabetes management as well as help clinicians in communicating with patients and improving intervention at the group level. Research has revealed that clinicians are currently the primary source of social support for patients with diabetes, with family members being a secondary source. Thus, for people with diabetes, their self-efficacy is affected by not only by their own actions but also the actions of clinicians. Few studies have focused on how interactions among patients with the same disease affect their self-efficacy. In the network development period, people with diabetes can readily acquire diabetes-related information from online diabetes groups. This has a potentially crucial effect on patients’ self-efficacy and diabetes self-management [31], and it can also increase the number of new channels for diabetes self-management, thus transforming the knowledge model mode of access for diabetes self-management from unidirectional to multidirectional. The aim of this study was to investigate participation in an online health community among people with diabetes, with a focus on how online interaction influences self-efficacy in the course of coping with diabetes. Other studies on diabetes self-management among people in diabetes online health communities have focused on the informational and technical support provided by online platforms and given less attention to individual cognitive and behavioral changes and the practical implications of online channels for diabetes health education [29,30]. Maybe diabetes patients with high self-efficacy are more likely to seek knowledge from the Internet than those with low self-efficacy. However, there are few studies to explain this problem. Moreover, different countries have different situations. Therefore, it is helpful to explore the relationship between self-efficacy and online interaction of Chinese diabetic patients, so we provide more evidence to illustrate the relationship between health self-efficacy and the intensity of online interaction. Thus, filling this research gap aids diabetes management by elucidating a new medical model that integrates aspects of physiology, psychology, and society [32,33].

## 2. Methods

### 2.1. Participants

Snowball sampling was applied to select users with diabetes or prediabetes who were active in a diabetes online health community. Data were collected through one-on-one online interviews with participants, building trust and answering participants’ questions during their completion of the questionnaire, and furthermore asking people with diabetes or prediabetes for their help in distributing the electronic questionnaires.

### 2.2. Instruments

This study measured the self-efficacy of patients with diabetes, along with those with pre-diabetes and those with critical-stage diabetes. The classical self-efficacy scale was modified and then tested. The scale combined behavioral characteristics with frequently discussed topics among Chinese people with diabetes in the online community. The 14-question diabetes self-efficacy scale developed by Lorig et al. for evaluating diabetes self-efficacy was modified and used to formulate the scale used in the present study. The valid and reliable diabetes self-efficacy scale developed by Grinslade et al. was also used by the developers of this scale [34,35].

Based on the diabetes self-efficacy scale developed outside of China, a novel electronic version of the Online Peer Education Survey for People with Diabetes was developed in this study. The contents of the scale are as follows: Demographic data (gender, age, place of birth, education level, area of residence, marital status), course of disease, blood glucose level, disease effect, use of diabetes online health communities, online interaction subscale (8 items; valid and reliable with Cronbach’s α = 0.676 > 0.600 and KMO = 0.794 > 0.700, respectively), motivation for group participation subscale (4 items; valid and reliable with Cronbach’s α = 0.872 > 0.800 and KMO = 0.826 > 0.800, respectively), self-efficacy subscale (13 items; valid and reliable with Cronbach’s α = 0.938 > 0.900 and KMO = 0.966 > 0.800, respectively), and online health community information dissemination factor subscale (15 items, including the strength of friendships made with other group members, information intensity, group professional intensity, recipient’s professional degree, and information collection; valid and reliable with Cronbach’s α = 0.916 > 0.900 and KMO = 0.958 > 0.900, respectively). Most of the abovementioned scales were graded on a 5-point Likert scale to evaluate a participant’s interaction in online health communities.

### 2.3. Procedure

The questionnaire administrators in this study were rigorously trained; they issued questionnaires on network platforms, such as QQ, WeChat, Tieba, forums, and diabetes-related apps. People with diabetes who met the inclusion criteria were invited to complete the questionnaire after obtaining their consent. The questionnaires were completed through one-on-one interviews using an online chat function. Questionnaire responses were then immediately examined manually on a professional platform named Wenjuanxing.

To ensure that the data were reliable and objective, questionnaire responses had to meet the following criteria to be included for analysis. First, respondents were required to satisfy the 1999 World Health Organization (WHO) diagnostic criteria for diabetes mellitus. The WHO criteria states a) that the people with diabetes are those with fasting blood glucose (FBG) >7.0 mmol/L or 2-h blood glucose after oral glucose tolerance test (OGTT) >11.0 mol/L, while the prediabetes are those with FBG = 6.1–7.0 mmol/L or impaired glucose tolerance = 7.8 mmol/L ≤ 2-h blood glucose after OGTT ≤ 11.1 mmol/L) and patients with diabetes at the critical stage have a FBG = 5.6–6.1 mmol/L. Second, respondents needed to have used a diabetes online health community for at least 1 week. Third, they must be volunteered to complete the electronic questionnaire. Fourth, respondents were required to be able to communicate normally with no history or present occurrence of mental illness. Fifth, respondents were required to be able to complete the questionnaire independently.

The exclusion criteria were as follows. The questionnaires were discarded of any two or more questionnaires with the same Internet protocol address. Questionnaires were also excluded if the time taken to complete the questionnaire was less than 200 s, logical errors or omissions were present in the questionnaire responses, the questionnaire answers were highly repetitive, or the respondents did not cooperate with investigators to examine the inquiries and supplementary instructions, except for those who were required to fill in the questionnaire correctly again.

#### Ethics Approval and Consent to Participate

All participants provided verbal informed consent to inclusion before they participated in the study, and the protocol was approved by the Ethics Committee of the Medical College of Shantou University (Code: SUMC-2016–39).

### 2.4. Data Analyses

This study assessed respondents’ self-efficacy level on the basis of their total score on the self-efficacy scale. A higher score indicated greater self-efficacy. Following reference to the literature, 13–25, 26–51, and 52–65 points were determined to be indicative of low, medium, and high self-efficacy, respectively [36].

**Figure 1:**
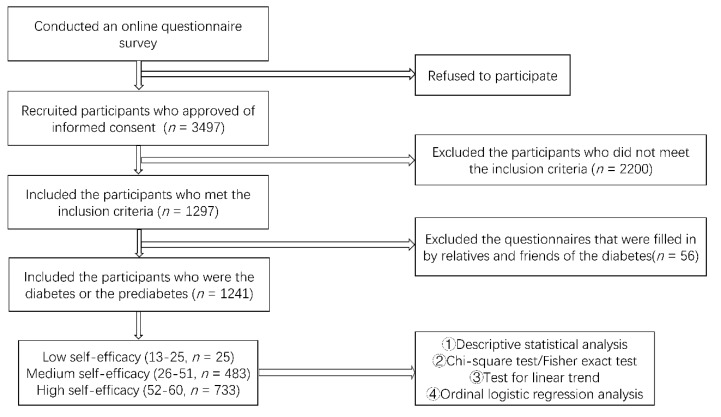
Research flow diagram.

SPSS (Version 22.0, IBM, New York, NY, USA) statistical analysis software and R software (Version 3.6.1) were used to analyze and visualize the questionnaire data, respectively. Descriptive statistics were used to describe the demographic characteristics, diabetes clinical characteristics, and online participation behavior of interviewees for each level of self-efficacy. Count data were described in terms of frequency and percentage. In a linear trend estimation, Chi-square test and Fisher’s exact were used to compare differences between the three groups and the Pearson correlation coefficient was used to determine the correlation between self-efficacy and engagement in diabetes online health communities. Chi-square test and Fisher’s exact were also used to analyze the interaction modes of respondents with different levels of self-efficacy. Ordinal logistic regression was used to examine the influence of various factors on the self-efficacy of respondents. Statistical significance was indicated if *p* < 0.05. The process of the whole study was demonstrated in Figure 1.

## 3. Results

### 3.1. Descriptive Statistical Analysis of Interviewed Participants with Diabetes or Pre-Diabetes

**Figure 2:**
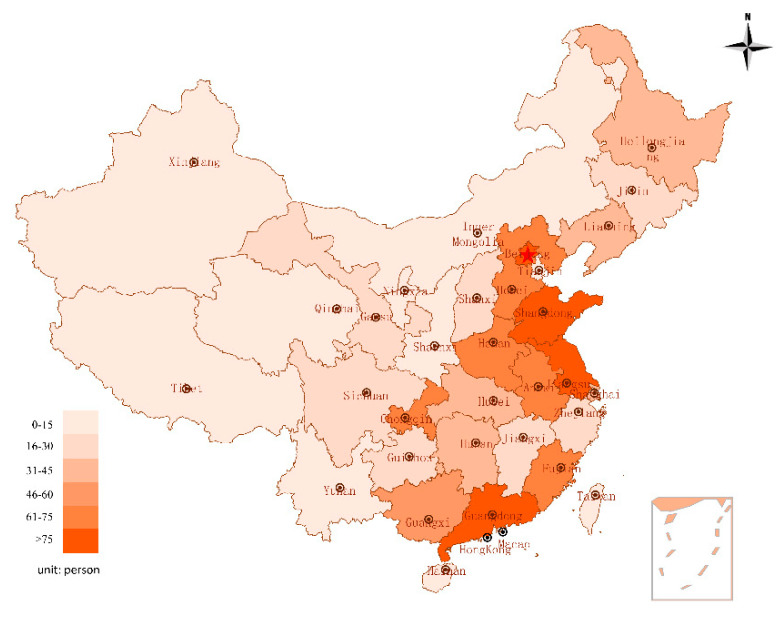
Distribution of participants’ region of residence.

**Figure 3:**
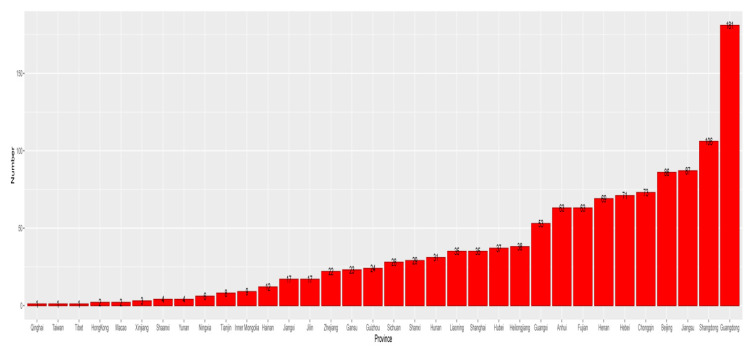
Participants’ provinces of residence.

In total, 1297 questionnaires were collected and met strict screening criteria, and 1241 questionnaires were deemed valid and used for analysis after including the participants who had diabetes and the prediabetes, and excluding the participants who filled in the questionnaires on behalf of their diabetic families, yielding an effective rate of 95.7%. After summarization and organization of the data, the mean score for self-efficacy ( $\bar{x}$ ± s) was determined to be 51.9 ± 9.12, which meant that most participants had a medium or higher level of self-efficacy. According to Shapiro Wilk test, *p* = 1.2333 × 10^−23^, which is less than 0.05, indicating that the total score data are a non normal distribution. According to the respondents’ IP addresses, most respondents were from 34 provinces, autonomous regions, and municipalities in China, with most coming from Guangdong province, and 12 respondents lived outside of China (see Figure 2). Because of the wide geographical distribution of participants, with each region represented by many participants, this study’s results are representative of all regions in China. Figure 3 maps the number of respondents for each region.

As indicated in Table 1, the gender ratio was approximately 1:1, and most respondents were 31–40 years old, holders of graduate or undergraduate qualifications, and urban residents (accounting for 63.1% of participants). Respondents’ diagnoses of diabetes were determined according to their blood glucose level, including for those with diabetes at the critical stage. Respondents in medium and high self-efficacy groups did not differ with respect to how long they had been members of the online diabetes group. Those who had been members for more than 1 year comprised 8%, 25%, and 25% of the low, medium, and high self-efficacy groups, respectively. Respondents viewed information in the online groups than they contributed information, with only 20.1% frequently (>10 times a day) posting information on the group. Respondents with medium and high levels of self-efficacy tended to more actively participate in larger groups; 73.1% of respondents were members of groups with more than 100 members. The most common (33.0% of all respondents) frequency of interaction with the group was 11–30 times per day. Most people (96% of all participants) perceived online diabetes groups to be useful for them.

### 3.2. Analysis of Self-Efficacy and Group Participation Situation of the Participants

Table 2 presents the results of a cross-analysis conducted to detect the difference of interaction modes among each subset of participants of different self-efficacy levels. The interaction modes were measured using eight items: “liking” posts, uploading or viewing pictures, reading and using group materials, viewing or responding to messages, viewing or sharing links, asking online-community friends for help, exchanging experiences or methods of treatment, and communicating with online-community friends through private messages. Chi-square test and Fisher’s exact test were conducted to compare statistical heterogeneity among the online interaction modes of the patients of each subgroup. The level of significance was α = 0.05. The results indicated significant differences between self-efficacy subgroups with respect to interaction modes (*p* < 0.05). Using a Bonferroni two-group comparison, we determined significant differences between respondents with medium self-efficacy and high self-efficacy with respect to interaction modes; participants with high self-efficacy adopted a greater variety of interaction modes than did their counterparts with medium self-efficacy (*p* < 0.0167), where those with low self-efficacy adopted the lowest variety of interaction modes. Low and medium self-efficacy subgroups significantly differed (*p* < 0.0167) with respect to frequency of viewing or responding to messages. Low and high self-efficacy subgroups significantly differed with respect to frequency of asking online-community friends for help and exchanging experiences or methods of treatment (*p*< 0.0167).

### 3.3. Analysis of Self-Efficacy and Demographic Characteristics and Online Participation of the Participants

Table 3 presents the results of the linear trend estimation for the relationships between self-efficacy and its antecedents; we noted significant linear relationships of self-efficacy level with marital status, frequency of viewing group information, time spent viewing community-provided information, and frequency of interaction with group members (*p* < 0.001). Results of a Pearson correlation analysis indicated that these relationships were positive (r > 0, *p*< 0.001) and that age and residential area were also positively related to self-efficacy level (r > 0, *p*< 0.01).

**Figure 4:**
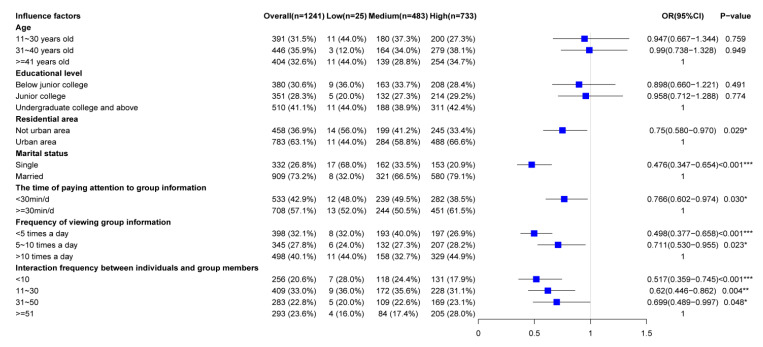
The forestplot of Ordinal logistic regression analysis results for influence factors and self-efficacy.

As is shown in Table 4 and Figure 4, this study’s dependent variable (“self-efficacy”) had three levels. Two binary logistic regressions were conducted to compare the following: (a) The low self-efficacy group with the medium and the high self-efficacy groups and (b) the low and medium self-efficacy groups with the high self-efficacy group. After the best factors had been selected by the above Chi-square test and Fisher’s exact test and confounders had been eliminated, the effects on self-efficacy from age, education level, residential area, marital status, time spent paying attention to group information, frequency of viewing group information, and frequency of interaction with group members were analyzed in an Ordinal logistic regression of a proportional odds model. The ordinal logistic regression equations were formulated according to the regression results. The equation used for the comparison of the low self-efficacy group with the medium and high self-efficacy groups was as follows.

ln(Rank = 1) = −5.221 + 0.288 × Residential area Not urban area + 0.742 × Marital status Single+ 0.266 × The time of paying attention to group information < 30 min/d + 0.697 × Frequency of viewing group information <5 times a day +0.340 × Frequency of viewing group information 5–10 times a day+ 0.659 × Interaction frequency between individuals and group members≤10 + 0.479 × Interaction frequency between individuals and group members11–30 + 0.359 × Interaction frequency between individuals and group members31–50(1)

The equation for the comparison of the low and medium self-efficacy groups with the high self-efficacy group was a cumulative probability model, as follows.

ln(Rank = 1) = −1.572 + 0.288 × Residential area Not urban area + 0.742 × Marital status Single+ 0.266 × The time of paying attention to group information < 30 min/d + 0.697 × Frequency of viewing group information <5 times a day +0.340 × Frequency of viewing group information 5–10 times a day+ 0.659 × Interaction frequency between individuals and group members≤10 + 0.479 × Interaction frequency between individuals and group members11–30 + 0.359 × Interaction frequency between individuals and group members31–50(2)

The results for the test of parallel lines cannot support the accuracy of the proportional odds model (*χ^2^* = 26.399, *p* = 0.009< 0.05). However, the sample was large, so the parallel lines test would be too sensitive to make *p* <0.05 even though the proportional advantage existed. The results for a Pearson goodness-of-fit test were *χ^2^* = 960.035 and *p* = 0.226 > 0.05, and those for a deviance goodness-of-fit test were *χ^2^* =826.301, *p*= 0.993 > 0.05; 755 cells (53.4%) also had a frequency of 0. The goodness-of-fit test results for the model demonstrated the model’s superiority relative to the model with only constant terms (*χ^2^* = 95.179, *p* < 0.05). The total prediction accuracy of the model can be obtained by calculation, that is, the number of predicted correct people in three groups divided by the total number of people = (143 + 596)/1241 = 59.5%

Respondents with higher self-efficacy were more likely to live in urban areas (*p* < 0.05) and be married (*p* < 0.05) and tended to spend more time paying attention to group information (*p* < 0.05), have a higher frequency of viewing group information (*p* < 0.05), and have a higher frequency of interaction with group members (*p* < 0.05).

### 3.4. Diabetes Information Sources for the Participants

**Figure 5:**
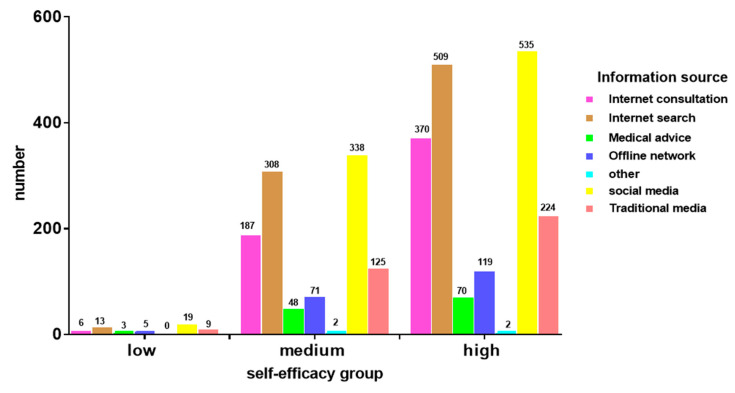
Multiple bar graph of diabetes information source in participants.

A cross-tab analysis of different levels of self-efficacy was conducted and the results are shown in Figure 5. The sources of information used by respondents were analyzed, with each source scored according to the number and percentage of respondents using it, in total and for each self-efficacy group. As indicated in Figure 5, the main sources of diabetes information for respondents of all self-efficacy levels were social media; 76.0% (19/25), 70.0% (338/483), and 73.0% (535/733) of respondents with low, medium, and high self-efficacy used this source, respectively. For all groups, advice from medical staff was rarely used as an information source; 12.0% (3/25), 9.9% (48/483), and 9.5% (70/733) of respondents with low, medium, and high self-efficacy used this source, respectively. The main sources of diabetes knowledge for respondents with low and medium self-efficacy were search engines and social media; those for respondents with high self-efficacy were search engines, social media, network platforms for professional advice, and mobile apps. Other sources of diabetes knowledge included scripture, magazines, and e-books.

### 3.5. Opinions of Interviewed Participants with Diabetes or Prediabetes Regarding Online Health Communities

After organizing and analyzing the questionnaire responses, this study screened the qualitative views of respondents who had been deeply influenced by their experience in online health communities.

#### 3.5.1. Female, 63 Years Old, Jiangsu-Nanjing, Diabetes Mellitus (ID: 70)

*“Because we are all patients with diabetes, we have compassion for each other, making us feel closer and helping us to communicate more easily. When I was sick, because of the small number of patients around me and the lack of Internet access, I found no patient like me to relate to for many years. Nobody could communicate with me or empathize with my feelings of helplessness. After joining an online diabetes community, I gained a lot of practical knowledge about blood glucose control that I couldn’t have gotten from books or even doctors. Talking in a group is not only enjoyable but also informative.”*

#### 3.5.2. Male, 48 Years Old, Tianjin, Diabetes Mellitus (ID: 78)

*“Different people have different methods of blood glucose control. I learned a lot from the group, and communicating with other people with diabetes felt really good. We had the same purpose: to learn how to better control our diabetes.”*

#### 3.5.3. Male, 21 Years Old, Jiangxi, High-Risk Population of Diabetes Mellitus (ID: 84)

*“For people who do not have enough self-control or who are not active enough in finding others like them, it is good to be part of a group where everyone can try to monitor and improve each other’s condition.”*

#### 3.5.4. Male, 35 Years Old, Henan, Diabetes Mellitus (ID: 144)

*“It has had a great effect on me. I have always felt inferior to others because of this disease. Other people in the online community give me comfort, which I found the most touching.”*

#### 3.5.5. Male, 29 Years Old, Henan-Jiaozuo, Diabetes Mellitus (ID: 162)

*“Seeing others share their experience of struggling with the illness has made me work harder and be more active in my life. The knowledge of blood glucose control shared by them has also helped me a lot. I really appreciate the moderator for allowing me to join this big family. Although I do not speak much, I have been paying attention to what goes on in the group, and I thank my friends with diabetes for giving me great courage in silence.”*

#### 3.5.6. Female, 45 Years Old, Shandong-Taian, Diabetes Mellitus (ID: 419)

*“At first, I felt a lot of pressure and negative emotions, but later, after communicating with other patients who could understand my situation, I slowly gained confidence. I would like to thank the online diabetes health community.”*

#### 3.5.7. Female, 38 Years Old, Heilongjiang-Harbin, Diabetes Mellitus (ID: 428)

*“I hope everyone is well. I have learned a lot in my half a year here. My life has been getting better. I will help new friends in the future, consult their professional knowledge, and be fearless.”*

#### 3.5.8. Female, 49 Years Old, Shanghai, Critical Diabetes Mellitus (ID: 519)

*“There are symptoms, topics, and concerns that we have in common. With mutual encouragement among people with diabetes, a bond forms; there is a feeling that they are more than just people who share a common experience.”*

#### 3.5.9. Male, 35 Years Old, Guangdong, Diabetes Mellitus (ID: 948)

*“People with diabetes are more accepting, and most of the information they share is credible. I have tried the methods they’ve recommended and they have been very useful.”*

#### 3.5.10. Male, 23 Years Old, Guangdong-Dongguan, Diabetes Mellitus (ID: 1097)

*“At present, my experience in online diabetes health communities still greatly influences me; it has been helpful to me. I know from this questionnaire which apps I can use to learn about diabetes.”*

For most respondents, their views on online diabetes health communities changed after joining them. Respondents reported several benefits from participation: they acquired health knowledge, improved their living habits, and were better able to cope with their emotional problems. Prior to joining the group, group members with diabetes experienced a much greater psychological burden and poorer quality of life. Upon joining the group, members gained greater mastery of the everyday skills required for diabetes self-management. When people with diabetes were in a bad mood, they had access to support from others with diabetes in the diabetes health communities. Through support from such a social network, community members gradually gained confidence when communicating and in their self-management of diabetes, greatly improving their quality of life and slowly increasing their self-efficacy.

## 4. Discussion

### 4.1. Chinese with Diabetes or Prediabetes Have Medium or High Self-Efficacy in Online Health Communities

Self-efficacy is an important factor affecting the control and quality of self-management of diabetes mellitus. The self-efficacy of the majority of the 1241 respondents to this study’s online questionnaire exceeded the high level. Adopting a classification of self-efficacy similar to that of this study, another study of patients with type 2 diabetes reported that 10.4%, 24.0%, and 65.6% of its participants had low, medium, and high self-efficacy, respectively, [36] which is a similar distribution to that of this study(2.0%, 38.9%, 59.1%).

Such high overall self-efficacy is attributable to the advantages of interactive education from an online community. First, with the development of a network and network tools, people with diabetes have become more independent in seeking informational support from the network. Online health communities also have the advantages of being convenient and providing immediate access to comprehensive information, especially regarding the various types of self-management for diabetes. With such communities, information dissemination is no longer confined to simple oral or paper-based media; information can be exchanged instantaneously, and patients and doctors in different regions can communicate remotely. Such convenience enables the provision of immediate and effective answers to questions, mitigation of negative emotions, and sharing of personal experience, thus better enabling people with diabetes to gradually develop confidence and improve their habits and approaches to disease self-management. Second, people with diabetes have common experiences. Specifically, they often worry about blood glucose control, the deterioration of diabetes, and complications from diabetes. Thus, these individuals can relate well to each other and have a shared emotional basis for communication. Moreover, in an online community, patients can discuss the pain and annoyance of elevated blood glucose levels, thus facilitating more emotional exchanges and the provision of emotional or material aid. When individuals with diabetes are heard by their peers, they develop a greater sense of identity and belonging, which improves their self-efficacy and forms a virtuous cycle [37,38].

Unlike traditional modes of health education, where information is provided from a single source, online diabetes groups are convenient to use; these groups enable timely communication and mutual assistance among people with diabetes as well as access to a wealth of information. Furthermore, online diabetes groups improve the emotional well-being of their users and allow experiences to be shared. These aspects improve the confidence and disease management of users with diabetes through interaction with their online peers.

### 4.2. Self-Efficacy Subgroups Significantly Differ in Respect to of Interactive Modes in Online Health Communities

In this study’s analysis, self-efficacy subgroups significantly differed with respect to modes of interaction in online communities for people with diabetes. These interaction modes included “liking” post, uploading or viewing pictures, reading and using community-provided information, viewing or responding to messages, asking online-community friends for help, exchanging experiences or methods of treatment, and communicating with online-community friends through private messages. Those with low and medium self-efficacy had less varied modes of interaction compared with those with high self-efficacy. This is attributable to the tendency of self-efficacy to promote an optimistic outlook, the willingness to help others and interact actively, and the confidence and aptitude for disease management through communication with others. By contrast, people with medium or low self-efficacy are less likely to benefit from participation in online diabetes groups. In general, people with high self-efficacy are more likely to participate in group interactions through a wide variety of interactive modes.

### 4.3. Most Diabetes-Related Information was Derived from Social Media

This may be related to the age distribution of the respondents; most were 11–40 years old. Social media is currently undergoing rapid development, and the free and convenient access to information that social media platforms provide contrasts with the cumbersome and expensive nature of medical treatments. Many studies on the information-seeking behavior of people with diabetes have demonstrated that young people often use the Internet to obtain health information and that the use of the Internet is correlated with a shorter diabetes duration [39]. Moreover, the Internet appears to be the main means by which young patients seek disease-related information.

Respondents with high self-efficacy were also more likely to live in urban areas and be married and tended to spend more time paying attention to group information, spend more time viewing group information, and have a greater degree of interaction with group members.

The participants who lived in urban areas tend to have higher self-efficacy, which might be caused by the economic condition for access to the network and knowledge reserve for using the network [40]. Married participants were more likely to have high self-efficacy. This is attributable to their reception of social support (in the form of encouragement, care, and supervision) from their family, which was the most important source of social support for all participants. Such social support promotes positive emotions and makes patients more resilient and determined to overcome obstacles [41].

The frequency of interaction between individuals and group members positively predicted self-efficacy. Because online health communities provide users with diabetes more opportunities for social interaction, users are more likely to encounter others who share their experiences. This reduces their sense of loneliness from having diabetes and aids the self-evaluation involved in their decision-making. These benefits entice people with diabetes to participate in online health communities, particularly to seek information [19]. Therefore, frequent interaction might be the potential factor for higher self-efficacy, which is consistent with the results of this study.

This study also determined that a higher frequency of viewing group information and longer time spent paying attention to community-provided information corresponded to higher self-efficacy. This is attributable to users having more access to sought information as well as more accurate and reliable information from these online groups.

This finding indicates that participation in online diabetes groups significantly improves self-efficacy in people with diabetes. Online diabetes education aided the blood glucose management of users with high self-efficacy. This study recommends a transition away from one-size-fits-all clinical interventions toward those that are tailored to individual characteristics and behaviors.

To our knowledge, the patients with high self-efficacy may tend to seek for health information online [42]. However, the behavior of seeking health information online is also directly mediated by reward assessment [43]. Reward assessment can refer to the attitude and evaluation on availability and helpfulness of the diabetes online health communities in the present study. We observed that the interaction in the diabetes online health communities brought benefit for the patients and their positive attitude toward these in the present study. Therefore, our study further validates and renews the relevant behavioral research model especially for the diabetes the first time in China. In addition, many studies just briefly state that the patients’ self-efficacy is influenced and changed by the intervention of the internet-based management, most are from the health care providers [44,45]. Our study can provide more evidence to fully prove the interrelationship between the self-efficacy and internet patient–patient interaction on the concrete aspects, that were the patients with high self-efficacy have ability and activation to search for information and then the strong connection of the peers will be developed. Conversely, the interaction in online health communities can positively predict the self-efficacy.

Furthermore, there is little evidence proved that the people with diabetes or prediabetes in China with high self-efficacy are more likely to seek information support from the Internet than those with low self-efficacy, so it is also meaningful for us to further verify this relationship. This study provided more evidence to clarify the relationship between health self-efficacy and online interaction intensity of people with diabetes or prediabetes in China. People with high self-efficacy have stronger practical ability and stronger ability to search information, so they have a relatively high intensity of online interaction. This study further verified and supplemented more evidence of people with diabetes or prediabetes in China for the first time. Because the evidence in different places and population may not be universal, and the results made in other places or population may not be representative enough to represent China’s specific medical culture, health culture, and the characteristics of the diabetes or prediabetes population in China (the largest population with diabetes or prediabetes in the world). Therefore, it is necessary to further study the relationship between health self-efficacy and Internet interaction intensity of the people with diabetes or prediabetes in China, in order to represent the online behavior characteristics of diabetes population in China.

### 4.4. Limitations and Expectations

Online surveys have some limitations, which must be addressed through the establishment of strict screening criteria. In this study, questionnaires were administered on an online platform to people with diabetes who met the inclusion criteria. However, because interaction between the researchers and respondents occurred online rather than in person, respondents were slow to develop trust in the researchers. Therefore, online surveys are easily ignored and distrusted, which makes the recovery of questionnaires more difficult. We recommend that online questionnaires be conducted in conjunction with the moderators of online health communities, especially with regard to their distribution, which would make it easier to motivate more people with diabetes to complete the questionnaires. In addition, to increase the reliability and rigor of the research results and reduce the influence of irrelevant variables (such as whether the respondents had diabetes and how long they had been members of the group), this study had defined inclusion criteria for participation. The interviewees should have enough experience in participating in the internet health community, and thus the patients without online community experience were not included. Moreover, interviewees’ responses may have been inconsistent with their true beliefs, whether as a result of unwillingness to report such beliefs or of inattentive reading of the questions, which would affect the robustness of the results. The trap questions were designed to identify respondents who completed the survey too quickly, who had reused the same IP address, or who gave duplicate answers. Because snowball sampling was used to recruit participants and participants were not supervised for the survey that was administered online, prospective respondents were more likely to fail the trap questions and respondents who passed these questions may not have completed the questionnaire with care, which reduced the validity of the questionnaire responses. Due to the lack of detailed investigations of people with diabetes, the findings of the present study must be further validated with respect to the diabetes self-management modes considered and the corresponding management results. With the development of the times, diabetes online social networking platform is a new means of self-management of diabetes. Diabetic patients are good at using these platforms, which can bring convenient and low-cost information, and it is a supplement to the previous diabetes management model. Among them, self-efficacy plays a bridge between diabetes patients and diabetes self-management. However, the accuracy of information provided by diabetes online social platform and the exact effect of diabetes self-management can be further tested. Furthermore, the influence of the participation of medical staff on the self-efficacy and self-management of diabetics can also be further explored.

## 5. Conclusions

An online diabetes health community is an integrated and interactive mode of interpersonal communication and information dissemination; it is constructed by people with diabetes for the purpose of exchanging knowledge and experience. This model is different from the traditional one-on-one mode of communication between doctors and patients. The key differences are as follows: (1) A difference in atmosphere: People with diabetes in online groups are more active and enthusiastic, they seek compassion from others with the same disease in a relaxed atmosphere or informal setting. Although medical staff can appropriately and comprehensively diagnose and treat the disease according to their protocol, they do so in a more serious atmosphere. (2) A difference in content: In online health communities, people with diabetes talk more about their experiences with self-management and medical consultation as well as various emotional and life challenges; these matters tend to be subtle and complex. By contrast, clinicians focus on discussing the examination, treatment, and prevention of diabetes in accordance with medical guidelines; patients are required to comply with doctor’s orders and strictly control and manage their own diseases. (3) A difference in impression: People with diabetes in online communities do not pay attention to their other identities. Most conversations emphasize a common problem. By contrast, medical staff may stereotype patients with diabetes as obese, sloppy, and sedentary, causing them to feel shame. (4) A difference in duration: Online communities offer longer-lasting support, thus providing people with diabetes with more opportunities to engage in communication. During doctor–patient communication, patients cannot fully express themselves because of time constraints. Doctors also dominate the interaction. (5) A difference in feedback: Online health communities have the advantages of enabling timely communication and the direct acquisition of information resources. By contrast, doctor–patient communication is limited by factors such as distance, money, and time. Moreover, doctor feedback is often inadequate and untimely. Despite its shortcomings, however, a doctor provides professional advice, which should provide a foundation for patients’ decisions when seeking medical treatment.

In this study, questionnaires were publicized through online diabetes health communities. This method of survey may be more popular among people with diabetes. Its advantages are as follows: (1) With the rapid development of network technology, more diabetes groups are being formed. Especially in present-day China, the popularity of QQ and WeChat groups provided a rich pool of respondents and improved this study’s coverage of the target population. (2) The electronic questionnaire did not require much explanation by the interviewer and could be directly understood and completed by the interviewee through the text prompt of the questionnaire; this reduced external interference. (3) In contrast to traditional paper-based surveys, electronic questionnaires can better protect the privacy of respondents, which is especially important for questionnaires regarding personal topics such as feelings of physical discomfort and mental health. (4) Electronic questionnaires are typically aesthetically pleasing and clearly displayed on a screen. Specifically, page transitions are automatic, and results can be automatically screened according to the respondent’s answers, and respondents are more receptive to such a format. (5) When organizing the results, no further manual input was required, thus limiting the potential for error.

Informational and emotional support from online health communities constitute a starting point for further research [46]. Understanding such support is crucial to revealing the mechanism that underlies how online involvement changes self-management behavior among people with diabetes. This finding indicates that a new direction in health education can strengthen the abilities of people with diabetes to collect and evaluate online information, especially that obtained from online communities, and interact with online users with diabetes. Thus, electronic health literacy can be improved by increasing the frequency of interaction between group members and changing the type of community-provided information to meet the expectations and demands of people with diabetes. Such information functions as requisite resources for promoting change in health behavior. These findings lay a theoretical foundation for healthcare workers in their practical conduction of health education through the use of network technology.

The findings of this study complement the literature on how online interaction in health communities affects self-efficacy and self-management among patients with diabetes mellitus. This study demonstrated the relationship between the intensity of online interactions and favorable diabetes self-care and self-efficacy. The findings imply that online interaction in health communities benefits people with diabetes with respect to the quality of self-care in a manner that hospital treatment cannot; such online interaction is thus an important supplement to the clinical treatment of diabetes mellitus. This study emphasizes the importance of diabetes self-management through online interactive education in the process of achieving optimal diabetes control. Furthermore, online interaction indirectly affects the quality of diabetes self-management through self-efficacy. To improve diabetes control by promoting self-efficacy, clinicians should consider encouraging patients to interact with other people with diabetes online; clinicians are aided when patients educate each other. In addition, in educating each other, better adherence to the practice of improving self-efficacy in diabetes management can be promoted. This ultimately enhances the benefits of patient–patient interaction with respect to ideal diabetes control.

## Figures and Tables

**Table 1:** The demographic characteristics and online participation of the participants.

Factors	Low (*n* = 25)	Medium (*n* = 483)	High (*n* = 733)	Overall (*n* = 1241)
Gender				
Female	8 (32.0%)	230 (47.6%)	333 (45.4%)	571 (46.0%)
Male	17 (68.0%)	253 (52.4%)	400 (54.6%)	670 (54.0%)
Age				
11–30 years old	11 (44.0%)	180 (37.3%)	200 (27.3%)	391 (31.5%)
31–40 years old	3 (12.0%)	164 (34.0%)	279 (38.1%)	446 (35.9%)
≥41 years old	11 (44.0%)	139 (28.8%)	254 (34.7%)	404 (32.6%)
Educational level				
Below junior college	9 (36.0%)	163 (33.7%)	208 (28.4%)	380 (30.6%)
Junior college	5 (20.0%)	132 (27.3%)	214 (29.2%)	351 (28.3%)
Undergraduate college and above	11 (44.0%)	188 (38.9%)	311 (42.4%)	510 (41.1%)
Residential area				
Not urban area	14 (56.0%)	199 (41.2%)	245 (33.4%)	458 (36.9%)
Urban area	11 (44.0%)	284 (58.8%)	488 (66.6%)	783 (63.1%)
Marital status				
Single	17 (68.0%)	162 (33.5%)	153 (20.9%)	332 (26.8%)
Married	8 (32.0%)	321 (66.5%)	580 (79.1%)	909 (73.2%)
Disease course				
<1 year	12 (48.0%)	201 (41.6%)	278 (37.9%)	491 (39.6%)
1–2 years	6 (24.0%)	131 (27.1%)	222 (30.3%)	359 (28.9%)
>2 years	7 (28.0%)	151 (31.3%)	233 (31.8%)	391 (31.5%)
Blood glucose				
High-risk diabetes	8 (32.0%)	78 (16.1%)	119 (16.2%)	205 (16.5%)
Critical diabetes	3 (12.0%)	198 (41.0%)	318 (43.4%)	519 (41.8%)
Diagnosed diabetes	14 (56.0%)	207 (42.9%)	296 (40.4%)	517 (41.7%)
Time to join groups				
<3 months	8 (32.0%)	114 (23.6%)	151 (20.6%)	273 (22.0%)
3–6 months	8 (32.0%)	128 (26.5%)	217 (29.6%)	353 (28.4%)
6–12 months	7 (28.0%)	108 (22.4%)	178 (24.3%)	293 (23.6%)
>1 year	2 (8.0%)	133 (27.5%)	187 (25.5%)	322 (25.9%)
Number of groups joined				
≤3	4 (16.0%)	192 (39.8%)	255 (34.8%)	451 (36.3%)
4–6	8 (32.0%)	169 (35.0%)	229 (31.2%)	406 (32.7%)
≥7	13 (52.0%)	122 (25.3%)	249 (34.0%)	384 (30.9%)
Frequency of viewing group information				
<5 times a day	8 (32.0%)	193 (40.0%)	197 (26.9%)	398 (32.1%)
5–10 times a day	6 (24.0%)	132 (27.3%)	207 (28.2%)	345 (27.8%)
>10 times a day	11 (44.0%)	158 (32.7%)	329 (44.9%)	498 (40.1%)
Frequency of sending group information				
<2 times a day	12 (48.0%)	248 (51.3%)	256 (34.9%)	516 (41.6%)
2–10 times a day	6 (24.0%)	158 (32.7%)	312 (42.6%)	476 (38.4%)
>10 times a day	7 (28.0%)	77 (15.9%)	165 (22.5%)	249 (20.1%)
The time of paying attention to group information everyday				
<30 min	12 (48.0%)	239 (49.5%)	282 (38.5%)	533 (42.9%)
>30 min	13 (52.0%)	244 (50.5%)	451 (61.5%)	708 (57.1%)
Size of groups with greater participation				
≤100	9 (36.0%)	127 (26.3%)	182 (24.8%)	318 (25.6%)
101–300	6 (24.0%)	136 (28.2%)	245 (33.4%)	387 (31.2%)
≥301	10 (40.0%)	220 (45.5%)	306 (41.7%)	536 (43.2%)
Interaction frequency between individuals and group members				
≤10	7 (28.0%)	118 (24.4%)	131 (17.9%)	256 (20.6%)
11–30	9 (36.0%)	172 (35.6%)	228 (31.1%)	409 (33.0%)
31–50	5 (20.0%)	109 (22.6%)	169 (23.1%)	283 (22.8%)
≥51	4 (16.0%)	84 (17.4%)	205 (28.0%)	293 (23.6%)
Help from group				
No help	6 (24.0%)	23 (4.7%)	21 (2.9%)	50 (4.0%)
A little help	9 (36.0%)	169 (35.0%)	172 (23.5%)	350 (28.2%)
General help	7 (28.0%)	178 (36.9%)	223 (30.4%)	408 (32.9%)
More help	3 (12.0%)	83 (17.2%)	188 (25.6%)	274 (22.1%)
A lot of help	0 (0%)	30 (6.2%)	129(17.6%)	159(12.8%)
Self-efficacy score				
Mean (SD)	20.8 (3.21)	44.6 (5.72)	57.8 (4.08)	51.9 (9.12)
Median [Min, Max]	22.0 [13.0, 25.0]	46.0 [26.0, 51.0]	57.0 [52.0, 65.0]	53.0 [13.0, 65.0]

SD: standard deviation; Min: minimum value; Max: maximum value.

**Table 2:** The cross analysis for self-efficacy and online group interaction mode of the participants.

Interaction Mode	Low (*n* = 25)	Medium (*n* = 483)	High (*n* = 733)	*χ* *^2^*	*p*
“Liking” posts				7.208	0.024 *
No	4 (16.0%)	93 (19.3%)	99 (13.5%)		
Yes	21 (84.0%)	390 (80.7%)	634 (86.5%)		
Uploading or viewing pictures				41.588	<0.001 ***
No	5 (20.0%)	152 (31.5%)	116 (15.8%)		
Yes	20 (80.0%)	331 (68.5%)	617 (84.2%)		
Reading and using group materials				34.337	<0.001 ***
No	6 (24.0%)	100 (20.7%)	67 (9.1%)		
Yes	19 (76.0%)	383 (79.3%)	666 (90.9%)		
Viewing or responding to messages				27.599	<0.001 ***
No	11 (44.0%)	100 (20.7%)	89 (12.1%)		
Yes	14 (56.0%)	383 (79.3%)	644 (87.9%)		
Viewing or sharing links				27.599	<0.001 ***
No	8 (32.0%)	118 (24.4%)	105 (14.3%)		
Yes	17 (68.0%)	365 (75.6%)	628 (85.7%)		
Asking online-community friends for help				20.711	<0.001 ***
No	8 (32.0%)	107 (22.2%)	96 (13.1%)		
Yes	17 (68.0%)	376 (77.8%)	637 (86.9%)		
Exchanging experiences or methods of treatment				39.313	<0.001 ***
No	9 (36.0%)	90 (18.6%)	59 (8.0%)		
Yes	16 (64.0%)	393 (81.4%)	674 (92.0%)		
Communicating with online-community friends through private messages				29.153	<0.001 ***
No	8 (32.0%)	171 (35.4%)	157 (21.4%)		
Yes	17 (68.0%)	312 (64.6%)	576 (78.6%)		

* *p* < 0.05, ** *p* < 0.01, *** *p* < 0.001.

**Table 3:** Test for linear trend for influence factors and self-efficacy of the participants.

Factors	Low (*n* = 25)	Medium (*n* = 483)	High (*n* = 733)	χ^2^	*P*1	r	*P*2
Gender				0.007	0.935	0.002	0.935
Female	8 (32.0%)	230 (47.6%)	333 (45.4%)				
Male	17 (68.0%)	253 (52.4%)	400 (54.6%)				
Age				9.561	0.002 **	0.088	0.002 **
11–30 years old	11 (44.0%)	180 (37.3%)	200 (27.3%)				
31–40 years old	3 (12.0%)	164 (34.0%)	279 (38.1%)				
≥41 years old	11 (44.0%)	139 (28.8%)	254 (34.7%)				
Educational level				2.877	0.009 **	0.048	0.009 **
Below junior college	9 (36.0%)	163 (33.7%)	208 (28.4%)				
Junior college	5 (20.0%)	132 (27.3%)	214 (29.2%)				
Undergraduate college and above	11 (44.0%)	188 (38.9%)	311 (42.4%)				
Residential area				11.121	<0.001 ***	0.095	0.001 **
Not urban area	14 (56.0%)	199 (41.2%)	245 (33.4%)				
Urban area	11 (44.0%)	284 (58.8%)	488 (66.6%)				
Marital status				41.078	<0.001 ***	0.182	<0.001 ***
Single	17 (68.0%)	162 (33.5%)	153 (20.9%)				
Married	8 (32.0%)	321 (66.5%)	580 (79.1%)				
Disease course							
<1 year	12 (48.0%)	201 (41.6%)	278 (37.9%)	1.165	0.280	0.031	0.280
1–2 years	6 (24.0%)	131 (27.1%)	222 (30.3%)				
>2 years	7 (28.0%)	151 (31.3%)	233 (31.8%)				
Blood glucose				0.267	0.606	−0.015	0.606
High-risk diabetes	8 (32.0%)	78 (16.1%)	119 (16.2%)				
Critical diabetes	3 (12.0%)	198 (41.0%)	318 (43.4%)				
Diagnosed diabetes	14 (56.0%)	207 (42.9%)	296 (40.4%)				
Time to join groups				0.86	0.354	0.026	0.354
<3 months	8 (32.0%)	114 (23.6%)	151 (20.6%)				
3–6 months	8 (32.0%)	128 (26.5%)	217 (29.6%)				
6–12 months	7 (28.0%)	108 (22.4%)	178 (24.3%)				
>1 years	2 (8.0%)	133 (27.5%)	187 (25.5%)				
Number of groups joined				2.272	0.132	0.043	0.132
≤3	4 (16.0%)	192 (39.8%)	255 (34.8%)				
4–6	8 (32.0%)	169 (35.0%)	229 (31.2%)				
≥7	13 (52.0%)	122 (25.3%)	249 (34.0%)				
Frequency of viewing group information				21.773	<0.001 ***	0.133	<0.001 ***
<5 times a day	8 (32.0%)	193 (40.0%)	197 (26.9%)				
5–10 times a day	6 (24.0%)	132 (27.3%)	207 (28.2%)				
>10 times a day	11 (44.0%)	158 (32.7%)	329 (44.9%)				
Frequency of sending group information				0.008	0.928	−0.003	0.928
<2 times a day	12 (48.0%)	248 (51.3%)	256 (34.9%)				
2–10 times a day	6 (24.0%)	158 (32.7%)	312 (42.6%)				
>10 times a day	7 (28.0%)	77 (15.9%)	165 (22.5%)				
The time of paying attention to group information				13.377	<0.001 ***	0.104	<0.001 ***
<30 min	12 (48.0%)	239 (49.5%)	282 (38.5%)				
>30 min	13 (52.0%)	244 (50.5%)	451 (61.5%)				
Size of groups with greater participation				0.008	0.928	−0.003	0.928
≤100	9 (36.0%)	127 (26.3%)	182 (24.8%)				
101–300	6 (24.0%)	136 (28.2%)	245 (33.4%)				
≥301	10 (40.0%)	220 (45.5%)	306 (41.7%)				
Interaction frequency between individuals and group members				21.177	<0.001 ***	0.131	<0.001 ***
≤10	7 (28.0%)	118 (24.4%)	131 (17.9%)				
11–30	9 (36.0%)	172 (35.6%)	228 (31.1%)				
31–50	5 (20.0%)	109 (22.6%)	169 (23.1%)				
≥51	4 (16.0%)	84 (17.4%)	205 (28.0%)				

* *p* < 0.05, ** *p* < 0.01, *** *p* < 0.001.

**Table 4:** Ordinal logistic regression analysis results for influence factors and self-efficacy.

Factors	Overall (*n* = 1241)	OR	*p*	OR 95% CI
Age				
11–30 years old	391 (31.5%)	0.947	0.759	0.667–1.344
31–40 years old	446 (35.9%)	0.990	0.949	0.738–1.328
≥41 years old	404 (32.6%)	1.000		
Educational level				
Below junior college	380 (30.6%)	0.898	0.491	0.660–1.221
Junior college	351 (28.3%)	0.958	0.774	0.712–1.288
Undergraduate college and above	510 (41.1%)	1.000		
Residential area				
Not urban area	458 (36.9%)	0.750	0.029 *	0.580–0.970
Urban area	783 (63.1%)	1.000		
Marital status				
Single	332 (26.8%)	0.476	<0.001 ***	0.347–0.654
Married	909 (73.2%)	1.000		-
The time of paying attention to group information				
<30 min/d	533 (42.9%)	0.766	0.030 *	0.602–0.974
≥30 min/d	708 (57.1%)	1.000		-
Frequency of viewing group information				
<5 times a day	398 (32.1%)	0.498	<0.001 ***	0.377–0.658
5–10 times a day	345 (27.8%)	0.711	0.023 *	0.530–0.955
>10 times a day	498 (40.1%)	1.000		-
Interaction frequency between individuals and group members				
≤10	256 (20.6%)	0.517	<0.001 ***	0.359–0.745
11–30	409 (33.0%)	0.620	0.004 **	0.446–0.862
31–50	283 (22.8%)	0.699	0.048 *	0.489–0.997
≥51	293 (23.6%)	1.000		-

* *p* < 0.05, ** *p* < 0.01, *** *p* < 0.001. OR: odds ratio; CI: confidence interval.
